# Return to active duty after anterior cruciate ligament reconstruction (ACLR) in Chinese male military aircrews

**DOI:** 10.3389/fsurg.2023.1232176

**Published:** 2023-08-25

**Authors:** Hongxing Zhang, Fengyuan Yang, Bowen Xie, Zhiqiang Chen, Ye Peng, Yufei Chen, Tianqi Li, Xiaogang Huang, Jing Xue, Junjie Du

**Affiliations:** ^1^ Department of Orthopedics, Air Force Medical Center of PLA, Beijing, China; ^2^ Air Force Clinical College, the Fifth School of Clinical Medicine, Anhui Medical University, Hefei, China; ^3^ Graduate School of Medicine, China Medical University, Shenyang, China

**Keywords:** anterior cruciate ligament injury, anterior cruciate ligament reconstruction, military aircrews, military medicine, postoperative recovery

## Abstract

**Background:**

Surgically treated anterior cruciate ligament (ACL) injuries may be a waivable condition and allow return to full flight status, but waivers are based on expert opinion rather than recent published data. The purpose of this study was to evaluate return to flight after anterior cruciate ligament reconstruction (ACLR) in male military aircrews with ACL injuries and to identify factors that affect flight clearance.

**Method:**

A single-center retrospective review was conducted by the authors for all active-duty aircrew who underwent ACLR at an authorized military medical center from January 2010 to December 2019. Demographic characteristics, occupational information, surgical data, and flight readiness evaluation outcomes were collected. Based on the final medical evaluation, subjects were divided into a qualified group (*N* = 64) and a disqualified group (*N* = 9), and the difference in data collected between the two groups was then analyzed to identify factors affecting flight clearance.

**Results:**

A total of 73 patients underwent successful ACLR with a mean age of 31.6 ± 5.6 years. Non-contact injury was the main type of ACL injury, accounting for 84.9% of the total injuries. 55 cases (75.3%) occurred during daily sports activities and 18 (24.7%) during military training. 64 of the 73 crewmembers (87.7%) were able to return to flight at their last follow-up evaluation. The preoperative interval time (PIT) was significantly less in the qualified group than in the disqualified group (*P* = 0.002). Patients who underwent ACLR within three months were more likely to return to flying than those who underwent the procedure three months later (97.4% vs. 76.5%, *P* = 0.010). The incidence of failure to return to flight duty was significantly higher in aircrews with ACL injuries combined with meniscal injuries than in aircrews with isolated ACL injuries (21.4% vs. 0.0%, *P* = 0.017).

**Conclusion:**

ACLR appears to be safe for military aircrew suffering ACL injuries with or without meniscal injury, and return to flight status is the most likely outcome for the majority of postoperative pilots. Prolonged PIT, PIT > 3 months, and ACL injury combined with meniscus injury had a negative impact on postoperative flight readiness.

## 1. Introduction

Anterior cruciate ligament (ACL) injury is one of the most common structural knee injuries (1) and occurs frequently in active young people and athletes, with studies showing that the incidence of ACL injury in the general population ranges from 0.3 to 0.68 cases per 1,000 person-years (2, 3). Military personnel may be at higher risk of ACL injury than the general population due to the rigorous physical demands of military training (4). The incidence of ACL injury in military personnel has been reported to be nearly ten times that of the general population, at 2.1 to 3.65 cases per 1,000 person-years (5, 6). For military aircrews, ever-increasing operational demands are placing greater demands on aircrew training and physical fitness, potentially increasing the risk of knee injuries among aircrews.

Anterior cruciate ligament reconstruction (ACLR) is designed to maintain knee stability and function (3). Although ACLR generally has a high success rate, post-operative pain and limited motion dysfunction can slow patients' reaction time or render them unable to perform certain necessary emergency procedures, resulting in permanent military flight restrictions or the end of their military careers, which ultimately affects the operational capability of military bases and strategic deployments. One of the most important indicators of aircrew's treatment in military medicine is the ability to return to flight status. To our knowledge, risk factors for athletic recovery and functional limitations after ACLR in athletes have been widely reported, but have not been extensively studied in the military population, and the impact of ACLR on military flight activities in aircrews has rarely been reported. The purpose of this study was to evaluate return to flight after anterior cruciate ligament reconstruction (ACLR) in military aircrews with ACL injuries and to identify factors that affect flight clearance.

## 2. Methods

### 2.1. Study design

The study was approved by the Ethics Committee of the Air Force Medical Center of the People's Liberation Army of China (PLA) (No. 2023-05-PJ01), informed consent was obtained from all patients, and was in accordance with the Helsinki Declaration. Chinese male aircrew who underwent ACLR surgery at the Air Force Medical Center from January 2010 to December 2019 were retrospectively reviewed, and final inclusion was determined based on the inclusion and exclusion criteria. The data collected included: (1) demographic information such as age, height, weight, body mass index (BMI), and smoking habit; (2) occupational data including service department, aircraft type, and total flight time; (3) injury and surgery-related information such as cause of injury, type of injury, preoperative interval time (PIT), whether combined with meniscus injury, and graft size. Each patient's electronic medical record was reviewed for return to flight status at 3 and 6 months postoperatively and at the final follow-up. For the purpose of this study, qualification was defined as successful return to flight duty and disqualification was defined as temporary or permanent grounding. The disposition of the patients at the last follow-up was recorded as a binomial distribution: qualified or unqualified for flight readiness.

### 2.2. Participants

The inclusion criteria for male participants were as follows: (1) patients hospitalized in our center from January 2010 to December 2019 who received treatment or adjusted flight status; (2) active military aircrews of the PLA Navy, Army, and Air Force (including pilots, navigators, communicators, mechanics, and other air combat personnel); (3) clinically diagnosed ACL injury patients who received surgical treatment. Subjects were required to meet all of the above criteria simultaneously.

The exclusion criteria for male participants were as follows: (1) patients hospitalized for treatment or flight status adjustment outside the study period; (2) non-active military flight personnel; (3) ground crews (including air traffic controllers, photographers, flight attendants, etc.); (4) unable to confirm time of injury; (5) patients with medical records less than 3 months old. Patients with any of the above conditions were excluded.

### 2.3. Flight qualification assessment

Irrespective of the treatment received, flight readiness must be assessed by the specialist surgeon at the authorized hospital in accordance with specific medical waiver guidelines. No pilot was granted the waiver unless the surgeon initially recommended clearance to return to flight status. The Military Pilot Specific Medicine Waiver Guide for Knee Injury was last updated in December 2022. The guidance requires a minimum ground observation period of 1 month after conservative treatment, 3 months after non-ligament reconstruction surgery and 6 months after ligament reconstruction surgery. Waivers will only be granted if the knee joint function is normal, or if the knee joint function is mildly restricted but does not affect daily life and ground work, physical fitness tests and rehabilitation assessments have been passed, and the joint surgeon has cleared the pilot to return to duty (Table 1). In addition, pilots of high-performance fighter aircraft must meet the requirements of a pedal test with a force of more than 120 kg maintained for more than 30 s. Currently, fighter and attack helicopter pilots with normal knee function after surgery, or mildly restricted knee function that does not interfere with daily life and ground work, may be granted flight waivers for dual-control aircraft (7).

**Table 1:** Knee function assessment scale.

Normal Knee Function (All of the following must be met)	Mildly Limited Knee Function (Meet all* of the following and 1 or more of the others)	Severely Limited Knee Function (Meet any 1 of the following)
No conscious pain, stiffness, or instability in the knee when resting, exercising, or exercising	Mild knee pain (VAS < 4) and/or mild swelling during or after exercise, without signs of locking or instability, and the pain or swelling is relieved by rest	More than moderate knee pain (VAS ≥ 4) and/or significant swelling during or after exercise, accompanied by stiffness and instability, and the pain or swelling does not resolve after 2 weeks of rest and symptomatic treatment.
Normal gait	Normal gait*	Abnormal gait
Bilateral thigh circumference difference <5%	Bilateral thigh circumference difference ≥5% but <10%	Bilateral thigh circumference difference ≥10%
Floating patella test (−)	Floating patella test (−)*	Floating patella test (+)
Patellar compression test (−) and patellar grinding test (−)	Patellar compression test (+) or patellar grinding test (+)	–
Knee joint stabilization	Knee joint stabilization*	Knee joint instability
The injured knee joint has no limitation to flexion and extension, and its active and passive flexion and extension activities are the same as the uninjured side	The injured knee joint is not restricted in extension, and the bilateral difference between active and passive knee flexion is <10˚	The injured knee joint is restricted in extension, or the bilateral difference between active and passive knee flexion is ≥10˚
Knee flexor and extensor strength (Grade Ⅴ)	Knee flexor and extensor strength (Grade Ⅴ)*	Knee flexor or extensor strength (less than Grade Ⅴ)
LSI ≥ 85% in any of the following: one-legged single jump, one-legged triple jump, one-legged cross jump, 6-meter timed one-legged jump	LSI < 85% in all of the following: one-legged single jump, one-legged triple jump, one-legged cross jump, 6-meter timed one-legged jump	–
Knee imaging is normal or shows no evidence of clinically significant changes	Knee imaging is normal or shows no evidence of clinically significant changes*	Knee imaging shows clinically significant changes

VAS, visual analogue scale; LSI, limb symmetry index.

* Key test items in knee function assessment.

### 2.4. Statistical analysis

SPSS software version 26.0 (SPSS Inc., Chicago, IL, United States) was used for data analysis. Measurement data were presented as means ± standard deviation or as absolute values with percentages (%). Data normality was assessed using the Kolmogorov-Smirnov test. Normally distributed data were evaluated using independent two-sample *t*-test, and chi-square test was used for non-normally distributed data. All *P* values were two-sided, and *P* < 0.05 was considered statistically significant.

## 3. Results

Table 2 shows the background data of the study participants. Between January 2010 and December 2019, a total of 73 military aircrews were included in the final study, with an average of 7.3 aircrews undergoing ACLR per year. These surgeries were performed by 5 different joint surgeons within the Orthopedics Department at the Air Force Medical Center, PLA. All patients were male with a mean age of 31.6 ± 5.6 years, mean height of 174.0 ± 3.7 cm, mean body weight of 74.1 ± 6.9 kg, mean BMI of 24.5 ± 2.3 kg/m^2^, and mean flying time of 1,458.4 ± 1,311.4 h. Of the 73 patients, 33 (45.2%) were younger than 30 years at the time of surgery, 31 (42.5%) were between 31 and 40 years of age, and the remaining nine (12.3%) were older than 40 years. By military branch, 16 (21.9%) served in the Army and 57 (78.1%) served in the Air Force. By aircraft type, 23 (31.5%) aircrews flew helicopters, 21 (28.8%) flew fighter aircraft, 12 (16.4%) flew trainer aircraft, ten (13.7%) flew transport aircraft, and seven (9.6%) flew other types of aircraft. Forty-four patients (54.8%) were nonsmokers and thirty-three (45.2%) were smokers.

**Table 2:** Baseline, injury, and surgery details for all participants and qualified and disqualified groups.

	Total cohort	Qualified group	Disqualified group	*P*-value
	(*N* = 73)	(*N* = 64)	(*N* = 9)	
Baseline data				
Age (year)	31.6 ± 5.6	31.3 ± 5.2	34.1 ± 7.5	0.154^a^
Height (cm)	174.0 ± 3.7	174.0 ± 3.8	174.0 ± 3.5	0.991^a^
Weight (kg)	74.1 ± 6.9	74.4 ± 6.8	72.2 ± 8.4	0.389^a^
BMI (kg/m^2^)	24.5 ± 2.3	24.6 ± 2.2	23.9 ± 2.8	0.398^a^
Flying time (hours)	1,458.4 ± 1,311.4	1,415.0 ± 1,268.5	1,766.9 ± 1,638.3	0.455^a^
Service				
Air Force (*n*)	57 (78.1%)	52 (91.2%)	5 (8.8%)	0.189^b^
Army (*n*)	16 (21.9%)	12 (75.0%)	4 (25.0%)	
Tobacco use				
Yes (*n*)	33 (45.2%)	28 (84.8%)	5 (15.2%)	0.758^b^
No (*n*)	44 (54.8%)	36 (90.0%)	4 (10.0%)	
Injury condition				
Cause of injury				
Military training (*n*)	18 (24.7%)	50 (92.6%)	5 (7.4%)	0.290^b^
Daily activities (*n*)	55 (75.3%)	14 (77.8%)	4 (22.2%)	
Injury type				
Contact (*n*)	11 (15.1%)	55 (93.2%)	7 (6.8%)	0.886^b^
Non-contact (*n*)	62 (84.9%)	9 (81.8%)	2 (18.2%)	
Meniscus injury				
Yes (*n*)	42 (57.5%)	33 (78.6%)	9 (21.4%)	**0**.**017**^b^
No (*n*)	31 (42.5%)	31 (100.0%)	0 (0.0%)	
Surgery information				
PIT (months)	7.5 ± 9.0	5.1 ± 4.9	24.3 ± 13.1	**0**.**002**^a^
≤3 months (*n*)	39 (53.4%)	38 (97.4%)	1 (2.6%)	**0**.**010**^b^
>3 months (*n*)	34 (46.6%)	26 (76.5%)	8 (23.5%)	
Graft size				
≤8 mm (*n*)	22 (30.1%)	18 (81.8%)	4 (18.2%)	0.541^b^
>8 mm (*n*)	51 (69.9%)	46 (90.2%)	5 (9.8%)	

^a^ Independent two-sample *t*-test was used.

^b^ Theoretical frequency ≥1 but <5 and continuously adjusted chi-square test was used.

BMI, body mass index; PIT, preoperative interval time.

Represented as means ± standard deviation or as absolute values with percentages (%). *P*-value for the difference between the qualified group and the disqualified group.

Significant values are shown in bold.

The injury and surgical characteristics of the subjects are also shown in Table 2. The results showed that non-contact injury was the main type of ACL injury, accounting for 84.9% of the total injuries. A total of 55 cases (75.3%) occurred during daily sports activities and 18 (24.7%) during military training. Basketball was the most common activity causing ACL injury, accounting for 54.5% of daily sports activities, followed by soccer (27.3%). The mean preoperative interval time (PIT) was 7.5 months, of which 34 patients (46.6%) were hospitalized and underwent surgery three months after injury, and 39 patients (53.4%) underwent ACLR within three months of injury. Admission MRI findings showed that 57.5% of ACL injuries were associated with meniscus injuries. Autologous hamstring tendon transplantation was performed in all patients who underwent surgery, with 69.9% of grafts larger than 8 mm.

**Figure 1:**
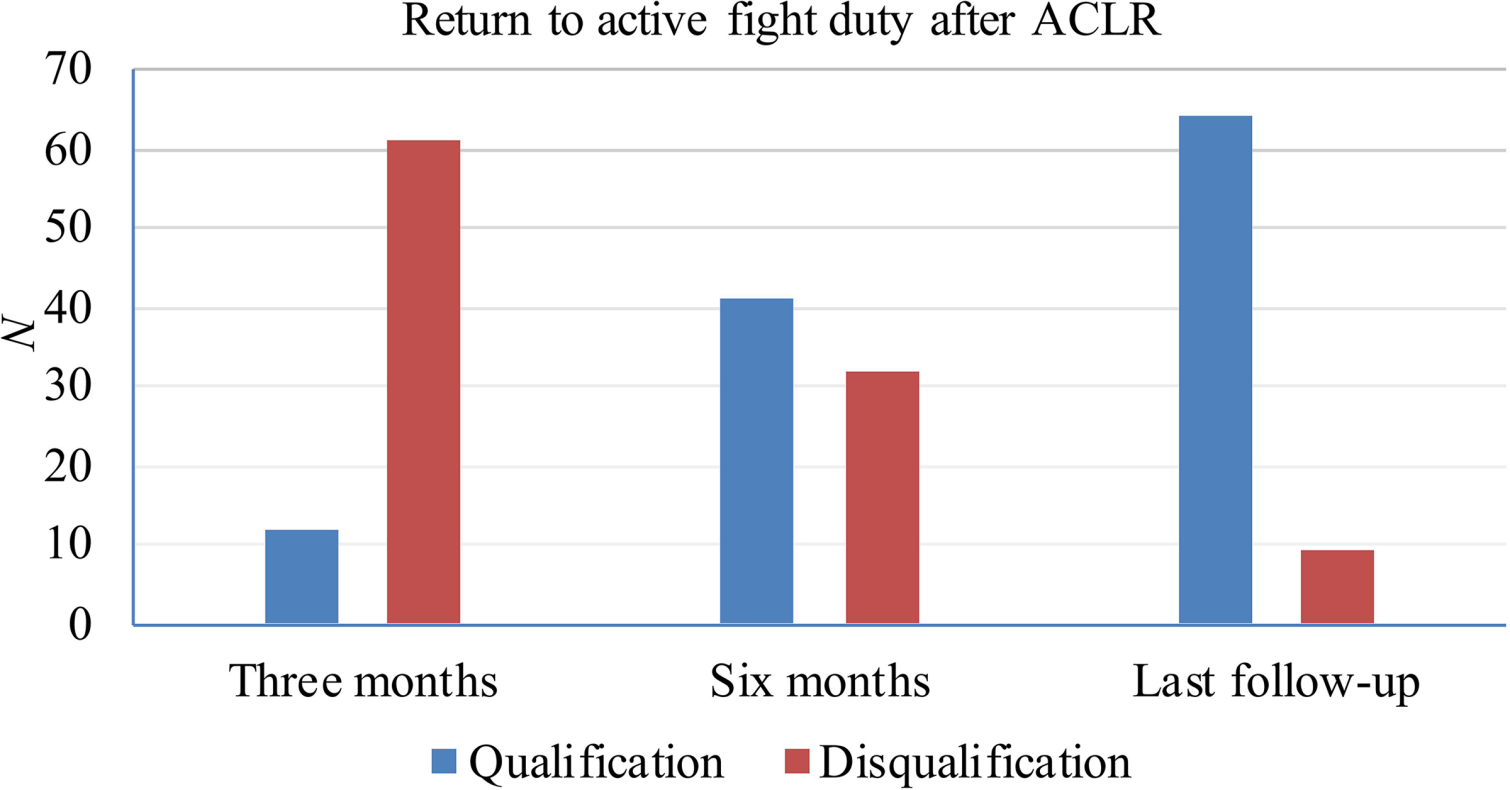
Military medical evaluation of aircrews s undergoing ACLR. “Qualification” means return to flight duty successfully, and “Disqualification” means temporary or permanent flight grounding.

After ACLR, all patients returned to our hospital for medical evaluation (Figure 1). Within three months of surgery, 12 patients (16.4%) returned to duty, while 61 patients (83.6%) were medically assessed as temporarily disqualified for flight and required to remain on ground observation or permanently removed from active flight status. At six months postoperatively, 41 (56.2%) had returned to duty and 32 (43.8%) were grounded. At the time of the last medical evaluation, 64 of the 73 aircrew (87.7%) were able to return to flight duty and 9 (12.3%) were still restricted from returning to flight duty. The mean postoperative follow-up time was 8.7 months.

As illustrated in Table 2, no significant differences in demographic and occupational data were found between the qualified (*N* = 64) and disqualified (*N* = 9) groups as classified by the final medical evaluation. The PIT was significantly less in the qualified group than in the disqualified group (*P* = 0.002). Patients who underwent ACLR within three months were more likely to return to flying than those who underwent the procedure three months later (97.4% vs. 76.5%, *P* = 0.010). In addition, the incidence of failure to return to flight duty was significantly higher in aircrews with ACL injuries combined with meniscus injuries than in aircrews with isolated ACL injuries (21.4% vs. 0.0%, *P* = 0.017). The qualified and disqualified groups did not differ significantly with respect to cause of injury (*P* = 0.290), injury type (*P* = 0.886), and graft size (*P* = 0.541).

## 4. Discussion

The purpose of this study was to evaluate return to flight after ACLR surgery in military aircrews with ACL injuries and to identify factors that affect flight clearance. The primary cause of ACL injuries to aircrews in the military environment is similar to that in the sports environment, and most occur during routine sports such as basketball and soccer rather than military training, with the most common mechanism of ACL injury being non-contact injury. This reflects that the daily physical training mode of aircrews is single and their sports protection is insufficient. Primary medical institutions should diversify the physical training methods of aircrews and strengthen the configuration of sports protection education and facilities. The intensity of aircrews’ physical training varies greatly from one type of aircraft to another. The training intensity and physical fitness activities of fighter and helicopter aircrews may cause injuries at a higher rate than other models. ACL injury occurred at about 30 years of age, which is the golden period of aircrews’ physical strength and performance of in-flight maneuvers. Flight suspension due to surgery has often been 6–12 months or more, which has a significant impact on both the personal development of aircrews and the military combat effectiveness of the Air Force. Marx et al. (8) found that 98% of surgeons would recommend surgery to a patient with an ACL injury, and 79% felt it would be impossible to resume all physical activity without ACLR. In addition, ACL injuries can lead to post-traumatic osteoarthritis at a relatively young age, which can result in flight disqualification and, in severe cases, termination of military service, resulting in a severe loss of military interest (9, 10). ACLR is considered the gold standard for treating ACL injuries in young athletes (11, 12).

A total of 73 aircrews underwent surgical treatment during the study period, all reconstructed using double-string autologous hamstring tendon grafts, and several studies showed that double-string ACLR with autologous hamstring tendon grafts tended to reduce the graft rupture rate and increase the probability of returning to pre-injury activity levels (13, 14). Thus, early ACLR may play an important role in restoring joint stability and reducing the risk of cartilage and meniscus damage (15, 16). While a few aircrews may not be able to regain normal knee function after ACLR due to potential knee pain, loss of quadriceps strength, loss of range of motion, and other biomechanical and functional abnormalities, the majority of those who undergo the procedure are eventually able to return to work successfully. This view was supported by our results, which showed that 64 of the 73 aircrews (87.7%) who underwent ACLR were medically cleared to return to work and only nine (12.3%) were permanently disqualified at the final medical evaluation. Anderson et al. (17) found that the cumulative probability of survival for service members with ACLR was 78.5% with at least four years of follow-up, which is slightly lower than our result. This may be due to the fact that Army soldiers are uniquely exposed to high physical demands, aggressive impacts, heavy loads, often on uneven terrain in suboptimal conditions compared to military aircrew. In contrast, a study of the impact of ACLR on the military careers of active-duty service members found that only 47.7% of service members returned to duty without restrictions and concluded that many service members cannot return to their official duties after ACLR and are permanently restricted (18). This difference may be explained by the female patients included in their study, as some studies have shown that female athletes have a poor prognosis in terms of return to sport after ACLR (19, 20). In addition, allografts, graft failure and subsequent secondary surgery potentially contributed to the low return to work rate.

Recent studies have identified several factors that negatively influence knee injury outcomes, including: increased BMI, active smoking, history of medial/lateral meniscus surgery, lateral meniscectomy, delay in primary ACLR, and global articular changes (17, 21). This study identified similar factors that had a negative impact on postoperative flight qualification including prolonged preoperative interval time (PIT) and ACL injury combined with meniscal injury. Therefore, early ACLR is recommended for patients with ACL injuries, and appropriate meniscal repair is required for the patients with combined meniscal injuries. In addition, many difficult clinical discussions surround the timing of surgery, which is a critical discussion point with a patient. Our study found that patients who underwent ACLR within three months were more likely to return to flying than those who underwent the procedure three months later. Possible reasons for this are that the delay in ACLR and its deleterious effects on the joint cause secondary injury leading to an increased risk of meniscal and cartilage damage (17, 20). Therefore, we suggest that three months post-injury is an appropriate time to discuss surgical timing and recommend that patients with ACL injuries undergo surgery as early as possible within this time frame.

In this study, the early return to flight rate after ACLR was ideal, but there may be some postoperative concerns to consider. Some studies have found that younger and more active individuals who participate in high-intensity exercise may be at particular risk for secondary ACL injury (22, 23). Young athletes who returned to sport within one year of ACLR were 15 times more likely to suffer a second ACL injury than the general population (24), and this risk of injury persisted for up to two years after return to sport, with the likelihood of a second ACL injury almost six times higher than in healthy control groups (16). An aircrew returning to flight is fundamentally different than returning to pre-injury training levels. This is because the post-operative patient may meet the requirements of the waiver guidelines and successfully return to flying, but may still not be able to fully adapt to high-risk military training, such as obstacle course training, or intense competitive sports activities, such as basketball and football.

Good communication between the orthopedic surgeon, physical therapist and flight surgeon is essential. The orthopedic surgeon is responsible for surgical outcomes and techniques, the physical therapist leads the rehabilitation decision making, and the flight surgeon provides supervision of the rehabilitation program. We recommend rehabilitation instruction immediately after ACLR, and patients discharged from the hospital will continue the rehabilitation program at their military post. The time-based rehabilitation protocols are mainly based on the remodeling process of the graft. Since there is still uncertainty about the timing of the human remodeling process and there are individual differences in neuromotor learning and flexibility after ACLR, it makes more sense to incorporate functional goal-based criteria into the rehabilitation protocol (25). After self-rehabilitation and self-evaluation, these patients should return to the hospital for a formal evaluation to determine if they can meet their goals for the next phase. Interventions in this phased rehabilitation program include cryotherapy, isometric quadriceps exercises, electrostimulation, closed and open kinetic chain training, bone-patellar tendon-bone and hamstring exercises, and neuromuscular training. Psychological interventions are sometimes necessary and can complement rehabilitation therapy, especially for patients with psychological and social stressors.

Early medical clearance after ACLR is mainly based on the above routine postoperative rehabilitation experience. Three months after surgery, some patients could resume their daily life and jogging, and could get on and off the airplane safely, so three months was taken into consideration as the time point for release. In recent years, based on the progress of the rule of tissue healing after ACLR, it was found that the plastic reconstruction period of the ligament graft began at 12 weeks (three months). Therefore, it is appropriate to complete the functional evaluation of the knee within 24 weeks (six months) after ACLR (26, 27). The existing medical waiver guideline suggests that the range of motion, strength, stability and flexibility of the knee joint can meet the requirements for safe flight about six months after surgery, therefore it is considered safe and appropriate to actually return to the flight post at this time. However, ligament reconstruction and shaping may take 1–2 years to complete, during which time the conditional application system of professional methods must be adopted, the rehabilitation of knee joint function must be promoted through the use of exercise equipment, and violent confrontational sports should be avoided for up to two years after surgery, and only then can motor function be assessed to evaluate whether the original activity level can be restored. In addition, studies have shown that current ACLR techniques are limited in their ability to prevent degenerative changes compared to non-surgical treatment (28). Long-term follow-up is needed to determine when aircrews can fully return to their pre-injury activities and postoperative knee degenerative changes following ACLR.

It must be said that the treatments for ACL injuries are only curative, and prevention should be considered the primary goal and deserves to be the focus of future research. Several intrinsic and extrinsic risk factors have been identified, including anatomical variations, neuromuscular deficits, biomechanical abnormalities, playing environment, and hormonal status (29). Patients with these risk factors should receive more attention for prevention. The lack of research on prevention programs for ACL injuries in the military community, particularly for military aircrew, can use the existing prevention program for the general population or athletes until military-specific research is available. Programs should include a combination of strengthening, stretching, aerobic conditioning, plyometrics, proprioceptive and balance training, and education and feedback regarding body mechanics and proper landing patterns (29). Multi-component prevention programs have been shown to be effective in reducing the incidence of this injury, but their success depends on implementation and compliance (1). Thus, researchers and clinicians must partner with military commanders, flight surgeons, service members (aircrews), and other stakeholders to identify barriers and strategies. Further research is needed to understand both the risk factors that contribute to ACL injuries and the mechanism by which ACL injury prevention programs are effective for this particular occupational group.

This study provides supplemental information on ACL injuries and evaluation in Chinese Air Force crews, and improves our understanding of military aircrew-specific medical waiver guidelines in bone and joint injuries for aeromedical researchers. However, the study still has some limitations that need to be considered. First, this study had a small sample size, which limited our ability to apply the results to the entire aircrew's population. Second, because of the short follow-up period, the long-term efficacy of our surgical method could not be evaluated. Third, there may be a selection bias because our population was limited to male flight crews. Prospective randomized controlled trials with large sample size, multicenter, and long-term follow-up are still needed to better evaluate the clinical efficacy of this procedure in the future.

## 5. Conclusions

ACLR appears to be safe for military aircrew suffering ACL injuries with or without meniscal injury, and return to flight status is the most likely outcome for the majority of postoperative pilots. Prolonged PIT, PIT > 3 months, and ACL injury combined with meniscus injury had a negative impact on postoperative flight readiness. Early ACLR is recommended for patients with ACL injuries, and appropriate meniscal repair is required for the patients with combined meniscal injuries.

## Data Availability

The raw data supporting the conclusions of this article will be made available by the authors, without undue reservation.
